# Effect of Warming on Growth, Grazing, and Community Composition of Free-Living Bacterioplankton in Subtropical Coastal Waters During Winter and Summer

**DOI:** 10.3389/fmicb.2020.534404

**Published:** 2020-10-06

**Authors:** Bowei Gu, Candy Lee, Xiao Ma, Yehui Tan, Hongbin Liu, Xiaomin Xia

**Affiliations:** ^1^Key Laboratory of Tropical Marine Bio-resources and Ecology, South China Sea Institute of Oceanology, Chinese Academy of Sciences, Guangzhou, China; ^2^University of Chinese Academy of Sciences, Beijing, China; ^3^Southern Marine Science and Engineering Guangdong Laboratory, Guangzhou, China; ^4^Department of Marine Science, The Hong Kong University of Science and Technology, Hong Kong, China; ^5^Hong Kong Branch of Southern Marine Science and Engineering Guangdong Laboratory, The Hong Kong University of Science and Technology, Hong Kong, China

**Keywords:** seasonal comparison, warming, grazing, bacterial community composition, bacterial abundance

## Abstract

Global warming is considered a major threat to marine ecosystems, which affects bacterioplankton activity, diversity, and community composition. However, few studies focus on the potential effects of warming on bacterioplankton in subtropical coastal waters in different seasons. Here we investigated the influences of warming on growth, grazing and community composition of bacterioplankton in Hong Kong coastal waters during winter and summer via 1-day incubation experiments. Our results revealed that without grazers, bacterioplankton displayed higher growth rate during summer compared to winter, while warming only significantly increased the growth rate of bacterioplankton in winter. Grazers with size <5 μm were major predators of bacterioplankton. Warming had little effect on grazing in summer but significantly enhanced grazing rates of >5 μm grazers in winter. In both seasons, warming had little influence on bacterial diversity and community composition. Nevertheless, in family and OTU levels, bacterioplankton had different responses to grazing and warming which may result from the selective grazing preference of predators and different temperature optima for bacterioplankton. Furthermore, the presence of >5 μm and <5 μm grazers would result in significant increase of some bacterial families under warming condition. Together, our results suggest that warming have direct impacts on bacterioplankton in subtropical coastal waters during winter and may thus affect global biogeochemical cycles.

## Introduction

Bacterioplankton are fundamental ingredients in marine ecosystems and play an important role in biogeochemical cycling (Azam et al., 1983; Cotner and Biddanda, 2002; Azam and Malfatti, 2007). Half or more of the total flux of matter and energy in the marine food web is thought to be pass through bacterioplankton, which make use of dissolved organic carbon released from viral lysis or grazing (Fuhrman, 1999). Recently, the significance of bacterioplankton that transform labile dissolved organic carbon to recalcitrant dissolved organic carbon is emphasized by microbial carbon pump theory which is a conceptual framework to address the processes and mechanisms for the generation of large ocean carbon reservoir (Jiao et al., 2010).

It’s widely accepted that predators control the standing stock of bacteria in global ocean (Fuhrman and Noble, 1995; Pernthaler, 2005; Kirchman, 2015). Around 40–95% of bacterivore is protist (Sanders et al., 2000; Zubkov and Tarran, 2008; Tsai et al., 2011). Nanoflagellates and ciliates, with size ranged from 1 μm to 200 μm, are recognized as major predators of bacterioplankton in various marine environments (Šimek et al., 1999; Christaki et al., 2001; Seong et al., 2006; Vargas et al., 2007; Jeong et al., 2008). Recent studies of grazing effects on natural bacteria have suggested that grazing affects not only the abundance of bacterioplankton but also the bacterial community composition via selective ingestion and digestion (Jürgens et al., 1999; Pernthaler, 2005; Gong et al., 2016). For example, Gong et al. (2016) showed that Alteromonadaceae, Pseudoalteromonadaceae, and Vibrionaceae are digestion-resistant bacteria, which could survive from protistan bacterivore feeding and accumulate in the community. It is generally believed that the size of bacteria is a factor that affects grazing (González et al., 1990; Posch et al., 2001). On the other hand, the grazing pressure can also trigger the change of the size structure of bacterial community (Jürgens et al., 1999). However, whether grazing would affect bacterioplankton community in subtropical waters remains unknown.

The dilution technique (Landry and Hassett, 1982) is a common method applied for quantifying the grazing effects of microzooplankton (Calbet and Landry, 2004; Schmoker et al., 2013). Nevertheless, several assumptions must be met to apply this technique appropriately. (i) prey growth rates must be independent of the dilution level. (ii) The ingestion rates of microzooplankton must be linearly proportional to the concentration of prey, meaning that microzooplankton are not food satiated. (iii) The changes in the density of prey over time follow an exponential model (Calbet and Saiz, 2013). Another method-size fractionation filtration, which is considered to have the least disturbance on the trophic webs has effectively evaluating the impacts of environmental changes on the dynamics of microbial communities (Zhang et al., 2007; Praadeep Ram and Sime-Ngando, 2008; Bouvy et al., 2011). This miniature field system is aimed to simplify nature to make it better understood instead of reproducing nature in an experimental model system (Bouvy et al., 2011).

Climate change such as ocean warming could have significant impacts on marine ecosystems (Wernberg et al., 2013). Model has predicted that the global temperature will be elevated exceed three degree till the end of this century (Rogelj et al., 2010). Warming may have significant impacts on bacterioplankton abundance and community composition (Pomeroy and Wiebe, 2001). It has been suggested that warming may increase bacterial losses to their grazers, and thus bacterial–grazer biomass flux within the microbial food web (Sarmento et al., 2010). Morán et al. (2015) found that warming results in a significant decadal trend of increasing bacterial abundance along with decreasing mean bacterial cell size. Moreover, recent studies observed that bacterial lineages response differently to warming due to different optimal temperatures (Pittera et al., 2014; Arandia-Gorostidi et al., 2017).

Abundance and community diversity of bacterioplankton display strong seasonal variations in seawaters (Bunse and Pinhassi, 2017). For example, in Hong Kong coastal waters, there is an order of magnitude difference in bacterial abundance between dry and wet seasons (Yuan et al., 2016). Our previous study also observed that Hong Kong coastal waters were dominated by Rhodobacteraceae during the summer time, while in winter it was SAR11 predominant (Xia et al., 2015a). In addition, seasonal variations in abundance and species of bacterial grazers in Hong Kong waters were observed (Chen et al., 2009). Therefore, we hypothesized that bacterioplankton community in subtropical waters would response differently to warming in different seasons. To test this hypothesis, incubation experiments were conducted using samples collected from Hong Kong coastal waters in December (winter) and September (summer). Bacterioplankton growth, grazing, and diversity in *in situ* temperature and elevated 3 degree were compared. Studying influence of increase of temperature on bacterial communities in different seasons allowed us to comprehensively evaluate the response of bacteria (abundance, growth, and diversity) to the global warming.

## Materials and Methods

### Incubation Experiment Setup

Surface seawater was collected from station PM7 (114°17.7′E, 22°20.4′N, eastern of Hong Kong), an ocean-influenced coastal station (Liu et al., 2018), using a 20 L polycarbonate (PC) carboy on December (winter, dry season) and September (summer, wet season). Water temperature and salinity were measured using a YSI EXO2 multiprobe sensor, which was calibrated before each sampling. Inorganic nutrient concentrations including nitrate, nitrite, ammonia, phosphate, and silicate were measured using a Skalar auto−analyzer (San Plus system, Netherlands) in the laboratory according to the JGOFS protocol.

For incubation, after prefiltration with 100 μm mesh, seawater was transported to the lab immediately (about 1 h) and then equally divided into three parts. One part was non-filtered (containing <100 μm grazers, hereafter <100 μm G group), the second part was filtered by 5 μm membranes (containing <5 μm grazers, hereafter <5 μm G group) and the last part was filtered by 1 μm membranes (nearly no grazers, hereafter NG group). Then each part was evenly distributed among 6 PC bottles (each bottle containing 250 mL seawater), and 3 of them were incubated in *in situ* temperature (samples from September: 28.5°C, samples from December: 19.1°C) and the other 3 bottles were incubated in a temperature 3°C higher than the *in situ* in an incubator (FIRSTEK, Taiwan, China) that has five independent enclosed shelves with different temperatures for 24 h. All bottles were incubated under the light intensity of approximately 100 μmol photons m^–2^ s^–1^ and a light: dark cycle of 14: 10. After incubation, 150 mL water from each bottle was prefiltered by 1 μm membrane and then filter through 0.2 μm membrane. Membranes were kept at −80°C until DNA extraction. Samples for flow cytometry analysis (1.8 mL) were also collected at initial and end of incubation. They were preserved with 0.5% buffered paraformaldehyde (v/v, final concentration), and stored in −80°C freezer until analysis.

### Flow Cytometry Analysis of Bacterial Abundance

Samples for bacterial abundance were analyzed by Becton-Dickson FACSCalibur flow cytometer (Becton Dickinson, San Jose, CA, United States). 0.01% SYBR Green I was added to stain the sample for 30 min in the dark at 37°C (Marie et al., 1997). Then, 1 μm fluorescent beads (Polyscience, Warrington, PA, United States) were added to each sample as an internal standard and samples were immediately analyzed at a flow rate of 0.25 μL/s for 1 min. WinMDI software 2.9 (Joseph Trotter, Scripps Research Institute, LaJolla, CA, United States) was used to analyzed the flowcytometric data.

### Bacterial Growth Rates and Grazing Rates by Grazers

An exponential growth rate (μ, day^–1^) was assumed for bacterioplankton in the absence of grazers (<1 μm fraction). The formula calculating net growth rate of bacterioplankton was as follows (Bouvy et al., 2011):

μ=(lnN-flnN)i/(t-ft)i

where N_*f*_ and N_*i*_ are the abundance of bacterioplankton at final (t_*f*_) and initial (t_*i*_) incubation time, respectively.

The grazing rate (*g*, day^–1^) of grazers was calculated using the same equation.

### DNA Extraction, PCR, and Pyrosequencing

Genomic DNA was extracted from the 0.2 μm membranes following a modified enzyme/phenol-chloroform extraction protocol (Xia et al., 2019). Briefly, the membranes were cut into small pieces (around 3 mm × 3 mm) with sterile scissors and transferred into 2 mL tubes with 0.5 mL of solution I (Tris–HCl 50 mM, EDTA 50 mM, and sucrose 50 mM; pH 8.0). After three freeze (−80°C)/thaw (60°C) cycles, lysozyme (5 mg mL^–1^, final concentration) was added to the tubes and incubated at 37°C for 1 h. Proteinase K (2 mg mL^–1^, final concentration) and sodium dodecyl sulfate (0.5%, w/v) were then added. After incubation at 60°C for 2 h, DNA in the solution was extracted once with 0.5 mL phenol-chloroform-isoamyl alcohol (25:24:1), and then twice with 0.5 mL of chloroform-isoamyl alcohol (24:1). The supernatants were then transferred to new tubes and 490 μL isopropyl alcohol (i.e., 70% volume of the supernatant) was added to each tube. After incubation at −20°C overnight, the tubes were centrifuged at 12,000 g for 10 min. The precipitated DNA samples were then washed twice using 0.2 mL 70% ethanol, and resuspended in 0.1 mL TE buffer (10 mM Tris–HCl, 1 mM EDTA, pH 8.0).

For pyrosequencing, the V3 and V4 regions of the 16S rRNA gene were amplified using primers 341F (5′-CCTAYGGGRBGCASCAG-3′), and 806R (5′-GGACTACN NGGGTATCTAAT-3′) (Beman et al., 2007). The PCR reaction was carried out in 25 μL master mix including 1.5 mM MgCl_2_, 1 × PCR buffer, 0.5 μM of each primer, 0.2 mM of each dNTP, 1.0 unit of Platinum^®^ Taq DNA polymerase (Invitrogen) and 1 μL genomic DNA. The PCR for each sample was carried out in triplicate with the following thermal cycles: 5 min initial denaturation at 95°C, followed by 30 cycles of 95°C for 30 s, 55°C for 30 s, and 72°C for 60 s, and then a final extension at 72°C for 7 min before holding at 4°C. The amplicons were then gel-purified using the Invitrogen gel purification kit (Invitrogen) and sequenced in Novagene company using an illumina Hiseq 2500 platform.

### Data Analysis

The 16S rRNA gene sequences were analyzed using the microbial ecology community software Mothur, following the standard operating procedure described by Schloss et al. (2009). To start, tags and primers were trimmed. Then sequences with an average quality score below 20 and lengths shorter than 300 bp were removed. Then sequences were aligned against the SILVA version 138 reference database. Columns where every character is either a “.” or a “−” were removed using the *filter.seqs* command. Chimeras were analyzed using the *chimera.uchime* command and removed. High quality sequences were identified using the Greengene version 13.8. database with cutoff value of 60% (Schloss et al., 2009). Sequences identified as chloroplasts, mitochondria or unknown, were removed. Remaining Sequences were clustered into Operational Taxonomic Units (OTUs) at cut-off values of 3%. Singletons (OTUs with just one sequence) were removed using the *remove.rare* command. Then 10,000 sequences were subsampled from each sample for subsequent analysis. To estimate similarity among the samples, Non-metric multidimensional scaling (NMDS) analysis and principal component analysis (PCA) were conducted based on the relative abundance of OTUs in each sample. OTUs were identified using the *classify.otu* command against the Greengene version 13.8. database (DeSantis et al., 2006).

We compared bacterial abundance and diversity index using Student’s *t*-test performed through SPSS statistical software. Significant difference (*P* < 0.01) between groups was assessed by analysis of similarities (ANOSIM) using PRIMER. The Global R, the analysis of similarities statistic represents separation degree of between-group and within-group mean rank similarities. *R* = 0 shows no separation while *R* = 1 indicates complete separation. The Statistical Analysis of Metagenomic Profiles (STAMP) software package was used to access significant differences between bacterial communities in each group (Parks et al., 2014). The relative abundance of the 30 most abundant bacterial OTUs, were log transformed and used to generate a heat map by HemI (Deng et al., 2014).

We predicted functional profiles from 16S rRNA gene data using the bioinformatic tool Tax4Fun (Aßhauer et al., 2015). Firstly, OTUs significantly response to the warming in each experiment were identified using the DESeq2 package (Love et al., 2014). Then, the OTUs were classified using SILVA database and the functional profiles were predicted using the Tax4Fun, which is subsequently normalized by the 16S rRNA copy number.

### Accession Numbers

All sequences obtained from this study have been deposited in the National Center for Biotechnology Information (NCBI) Sequence Read Archive under accession number: PRJNA596599.

## Results

### Effect of Warming on the Abundance, Growth, and Grazing of Bacterioplankton

**FIGURE 1:**
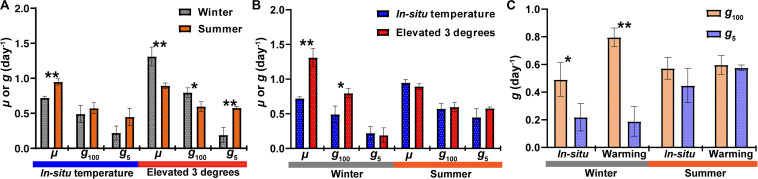
The response of bacterial growth rate (μ) and grazing rate (*g*) to warming in winter and summer. **(A)** Comparisons of μ and *g* between seasons. **(B)** Comparisons of μ and *g* between incubations in *in-situ* temperature and elevated 3 degrees. **(C)** Comparisons of *g* between different size grazers. *g*_100_, grazing rate of <100 μm grazers; *g*_5_, grazing rate of <5 μm grazers. * represents there are significant differences between them (Student’s *t*-test; **P < 0.01; *P < 0.05).

In Hong Kong waters, bacterial abundance was around 6.5 × 10^5^ cells mL^–1^ in winter and 9.9 × 10^5^ cells mL^–1^ in summer (Supplementary Figure S1). In both seasons, after 24 h incubation, bacterial abundance increased in all groups. Compared with *in situ* temperature incubation, bacterial abundance increased markedly in warming treatment during winter, especially in the 5 μm-prefiltration (W- <5 μm Gt) and non-grazer treatment (W-NGt), which increased from 0.99 ± 0.11 × 10^6^ cells mL^–1^ and 1.22 ± 0.03 × 10^6^ cells mL^–1^ to 1.83 ± 0.17 × 10^6^ cells mL^–1^ and 2.21 ± 0.30 × 10^6^ cells mL^–1^, respectively. No statistical difference of bacterial abundance in response to warming was found during summer (Supplementary Figure S1 and Table 1). In contrast, warming resulted in tremendous increase of bacterial abundance in winter whereas had no effect on bacterial abundance in summer. As shown in Supplementary Figure S1 and Table 1, bacterial abundance of non-grazer treatment (W-NG) was around 1.22 ± 0.03 × 10^6^ cells mL^–1^, significantly higher than that of 100 μm-prefiltration treatment (W- <100 μm G, Student’s *t*-test, *P* = 0.001) and 5 μm-prefiltration treatment (W- <5 μm G, Student’s *t*-test, *P* = 0.024) in winter. Nevertheless, there was no significant difference of bacterial abundance between W- <100 μm G and W- <5 μm G (Student’s *t*-test, *P* = 0.053, Table 1). Similar pattern was observed in experiment conducted in summer (Student’s *t*-test, *P* = 0.17 between S- <100 μm G and S- <5 μm G; *P* < 0.001 between S- <100 μm G and S-NG; *P* = 0.002 between S- <5 μm G and S-NG; Supplementary Figure S1 and Table 1). Without grazers, bacterioplankton displayed higher growth rate in summer (μ = 0.95 ± 0.04 day^–1^) compare to winter (μ = 0.72 ± 0.02 day^–1^), while warming only significantly increased the growth rate in winter (Figure 1). In summer, the grazing rates of <100 μm grazers and <5 μm grazers were 0.57 ± 0.08 day^–1^ and 0.45 ± 0.12 day^–1^, respectively, which may infer that grazers with size <5 μm were major predators of bacterioplankton in summer. There was no significant difference of grazing rates between winter and summer whereas this difference become significant in warming treatment (Figure 1). Warming merely significantly promoted the grazing rate of <100 μm grazers in winter which indicated that warming could strengthen the grazing effect of grazers lager than 5 μm in winter and have little effect on the grazing of grazers smaller than 5 μm in both seasons (Figure 1).

**TABLE 1:** The *P* values of Student’s *t*-test for the difference of bacterial abundance and diversity index between each group.

	Abundance		ACE		Shannon		Simpson	
Group	Winter	Summer	Winter	Summer	Winter	Summer	Winter	Summer
100G vs. 5G	0.053	0.17	0.354	0.364	0.819	0.562	0.991	0.473
100G vs. NG	**0.001**	**<0.001**	0.543	0.648	0.182	0.703	0.499	0.269
5G vs. NG	**0.024**	**0.002**	0.752	0.419	0.116	0.397	0.502	0.056
100G vs. 100Gt	**0.024**	0.173	0.213	0.305	0.716	0.068	0.452	0.059
5G vs. 5Gt	**0.002**	0.079	0.882	**0.029**	0.359	0.593	0.307	0.19
NG vs. NGt	**0.005**	0.179	0.893	0.386	0.841	0.223	0.419	0.21
100Gt vs. 5Gt	**0.001**	0.715	0.586	0.386	**0.002**	0.073	0.117	0.291
100Gt vs. NGt	**0.002**	**<0.001**	0.332	0.096	0.2	**0.02**	0.13	**0.049**
5Gt vs. NGt	0.125	**<0.001**	0.688	**0.026**	0.113	0.349	0.104	0.391

*100G, <100 μm grazers; 5G, <5 μm grazers; NG, no grazers. 100G, 5G, NG groups were incubated in *in situ* temperature while 100Gt, 5Gt, NGt groups were incubated in elevated three degrees. *P* < 0.05 were bold.*

### Effect of Warming on Bacterial Community Composition and Functional Profiles

**FIGURE 2:**
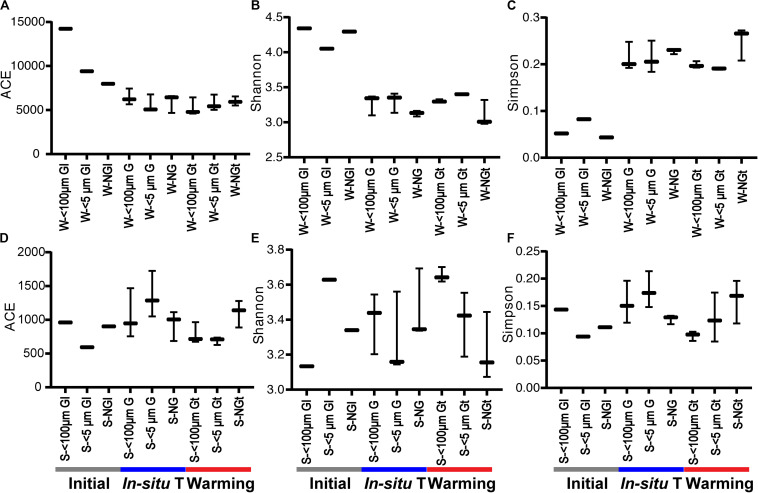
The diversity indices of bacterial community in each experiment group during winter **(A–C)** and summer **(D–F)**. Initial, the ambient samples; *In situ* T, incubation in *in situ* temperature; Warming, incubation in the elevated three degrees <100 μm G, only containing grazers smaller than 100 μm; <5 μm G, only containing grazers smaller than 5 μm; NG, no grazers. W, winter; S, Summer. GI, initial groups; G, groups incubated in *in situ* temperature; Gt, groups incubated in temperature three degrees higher than the *in situ*.

The values of the diversity indices in winter were higher than that in summer. Generally, warming had little effect on bacterial community diversity, except significantly deceasing the ACE index (representing community richness) of 5 μm-prefiltration treatment (S- <5 μm Gt) in summer (Figure 2).

**FIGURE 3:**
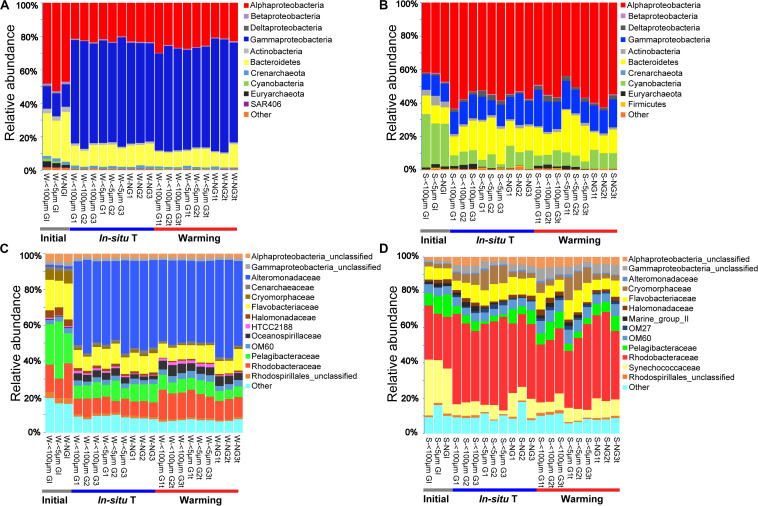
Bacterial community composition of each experiment sample as relative abundances at phylum **(A,B)** and family **(C,D)** level during winter **(A,C)**, and summer **(B,D)**. Initial, the ambient samples; *In situ* T, incubation in *in situ* temperature; Warming, incubation in the elevated three degrees. <100 μm G, only containing grazers smaller than 100 μm; <5 μm G, only containing grazers smaller than 5 μm; NG, no grazers. W, winter; S, Summer. GI, initial groups; G, groups incubated in *in situ* temperature; Gt, groups incubated in temperature three degrees higher than the *in situ*.

In total, 42 and 32 bacterial phyla were detected among all samples in winter and summer, respectively, although most of them had a relative abundance less than 1.0%. Proteobacteria contributed 62.1–89.3% of total bacterial phyla in winter and 52.0–78.4% in summer, ranked as the most abundant bacterial phylum. The relative abundance of Proteobacteria for each experiment group varied slightly both in winter and summer (Figure 3). Two predominant class of Proteobacteria were Alphaproteobacteria and Gammaproteobacteria. The second abundant phylum was Bacteroidetes, contributed 8.2–25.5% in winter and 10.4–25.2% in summer. Cyanobacteria was the third abundant phylum in summer whereas it made little contribution in winter. It was indicated that bacterial phylum showed little responses to warming in both seasons (Figure 3).

**FIGURE 4:**
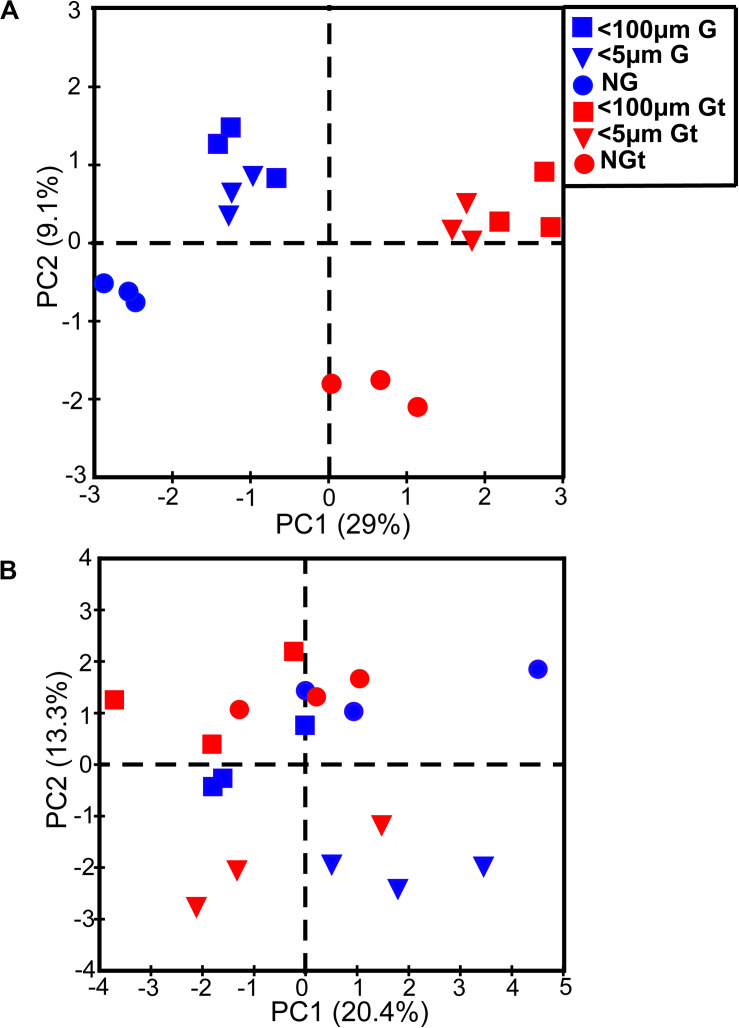
Principle component analysis (PCA) showing bacterial community composition of each experiment sample in winter **(A)** and summer **(B)**. Blue, samples incubated in *in situ* temperature; Red (t), samples incubated in the elevated three degrees. <100 μm G, only containing grazers smaller than 100 μm; <5 μm G, only containing grazers smaller than 5 μm; NG, no grazers. G, groups incubated in *in situ* temperature; Gt, groups incubated in temperature three degrees higher than the *in situ*.

There was a large difference between bacterial communities for each incubation groups with a high global *R*-value in the ANOSIM tests in winter (Global *R* = 0.798, *P* = 0.001) while a slight but significant difference in summer (Global *R* = 0.337, *P* = 0.001; Supplementary Figure S2). Interestingly, bacterial communities which were incubated in *in situ* temperature and in elevated temperature were separated in winter whereas clustered closely in summer (Supplementary Figure S2). PCA with first two axes explained 38.1% variances in winter and 33.7% in summer (Figure 4) revealed similar pattern than NMDS analysis. However, ANOSIM tests show there was no significant difference between groups incubated in *in situ* temperature and elevated three degrees which may reflect warming had little effect on bacterial community composition in both seasons (Table 2).

**TABLE 2:** ANOSIM tests showing the difference of bacterial community composition between individual two groups.

	Winter		Summer	
Group	R	*P*	R	*P*
100G vs. 5G	0.333	0.1	0.407	0.1
100G vs. NG	0.481	0.1	0.259	0.1
5G vs. NG	0.852	0.1	0.185	0.4
100G vs. 100Gt	0.963	0.1	0.222	0.4
5G vs. 5Gt	0.852	0.1	0.185	0.2
NG vs. NGt	0.926	0.1	0.037	0.4
100Gt vs. 5Gt	0.444	0.1	0.741	0.1
100Gt vs. NGt	0.778	0.1	0.63	0.1
5Gt vs. NGt	0.704	0.1	0.63	0.1

*100G, <100 μm grazers; 5G, <5 μm grazers; NG, no grazers. 100G, 5G, NG groups are incubated in *in situ* temperature, while 100Gt, 5Gt, NGt groups are incubated in elevated three degrees.*

**FIGURE 5:**
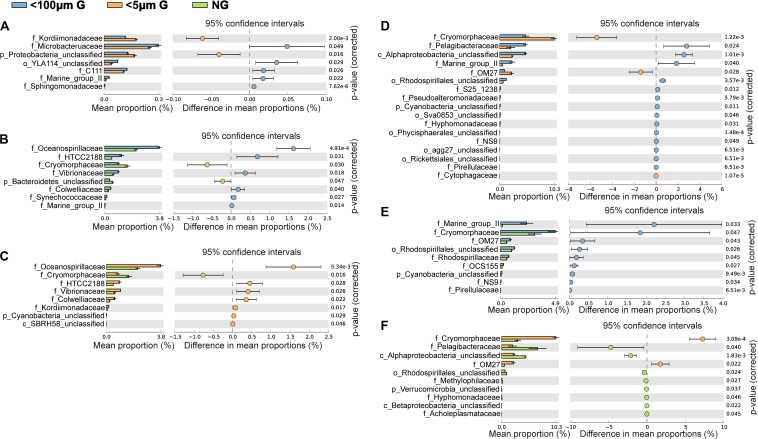
Statistical Analysis of Metagenomic Profiles (STAMP) analysis of the relative abundance of the bacterial families significantly enriched or depleted between the 100 μm-prefiltration **(A,D)**, 5 μm-prefiltration **(B,E)** and 1 μm-prefiltration **(C,F)** treatment in winter **(A–C)** and summer **(D–F)**.

**FIGURE 6:**
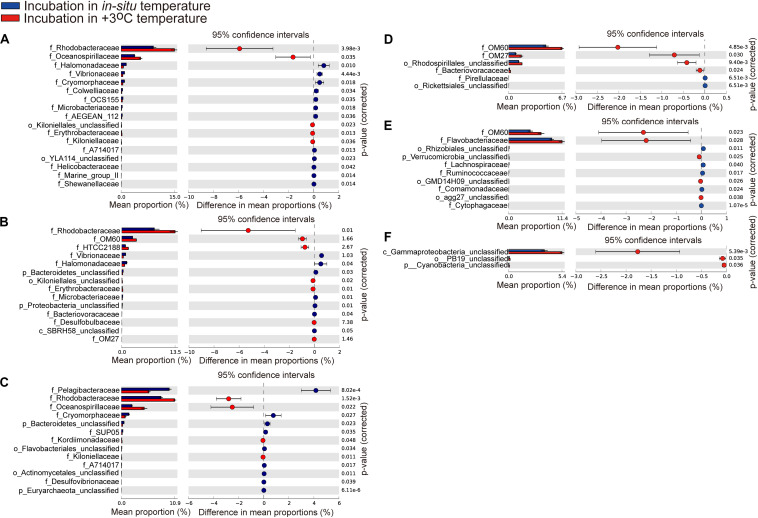
Statistical Analysis of Metagenomic Profiles (STAMP) analysis of the relative abundance of the bacterial families for 100 μm-prefiltration **(A,D)**, 5 μm-prefiltration **(B,E)**, and 1 μm-prefiltration **(C,F)** treatment significantly enriched or depleted between the *in situ* temperature and elevated three degrees treatment in winter **(A–C)** and summer **(D–F)**.

**FIGURE 7:**
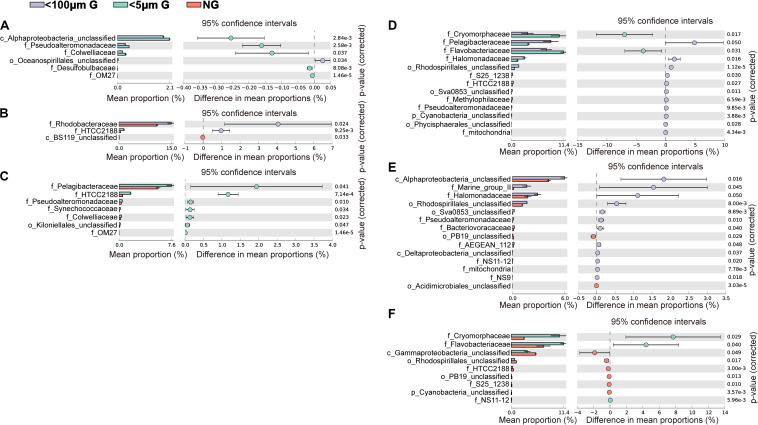
Statistical Analysis of Metagenomic Profiles (STAMP) analysis of the relative abundance of the bacterial families significantly enriched or depleted between the 100 μm-prefiltration **(A,D)**, 5 μm-prefiltration **(B,E)** and 1 μm-prefiltration **(C,F)** treatment after warming in winter **(A–C)** and summer **(D–F)**.

At the family level, the most abundant family in the *in situ* surface water of PM7 was Pelagibacteraceae in winter with a relative abundance of 16.9–31.8%, which together with that of Cenarchaeaceae, Flavobacteriaceae, Halomonadaceae, and Rhodobacteraceae decreased during incubation (Figure 3C). In contrast, the relative abundance of Alteromonadaceae dramatically increased after incubation (Figure 3C). In summer, Rhodobacteraceae was the most abundant family with a relative abundance of 26.2–30.5%. Synechococcaceae, which was the second abundant family (24.3–31.7% relative abundance) decreased after incubation along with Pelagibacteraceae, contrary to Alteromonadaceae, Cryomorphaceae, Flavobacteriaceae, and Rhodobacteraceae. The families of bacteria exhibited different responses to grazing (Figure 5). For instance, Oceanospirillaceae, HTCC2188, Vibrionaceae, Colwelliaceae, Synechococcaceae and Marine group II were positively affected by >5 μm grazers, while Cryomorphaceae was negatively affected by >5 μm grazers (Figure 5C). In summer, Cryomorphaceae and OM27 were significantly enhanced by <5 μm grazers and weakened by >5 μm grazers while Pelagibacteraceae and Hyphomonadaceae showed the opposite patterns (Figures 5D,F). Meanwhile, the bacterial families displayed distinct responses to warming as well (Figure 5). In winter, due to the elevated temperature, 17, 14, and 13 families were shown to achieve a significant difference (*P* < 0.05) in <100 μm G, <5 μm G and NG groups, respectively (Figure 6). Rhodobacteraceae was significantly enhanced by warming while Pelagibacteraceae was significantly weakened by warming without grazers in winter (Figure 6). In contrast to the warming effect on bacterial families in winter, that in summer was in general minor. Only 6, 10 and 3 families were observed to make a statistically difference (*P* < 0.05) in <100 μm G, <5 μm G and NG groups in parallel (Figure 6). Most of these families were generally positively influenced by warming in all groups, such as OM60 and OM27 in <100 μm G group, OM60 and Flavobacteriaceae in <5 μm G group and unclassified affiliated to Gammaproteobacteria in NG group (Figures 6D–F). In warming treatment, bacterial families revealed different responses to grazing both in winter and summer (Figure 7). For example, unclassified affiliated with Oceanospirillales was generally positively affected by >5 μm grazers while Pesudoalteromonadaceae, OM27, etc. were generally negatively affected by >5 μm grazers in winter (Figure 7A).

**FIGURE 8:**
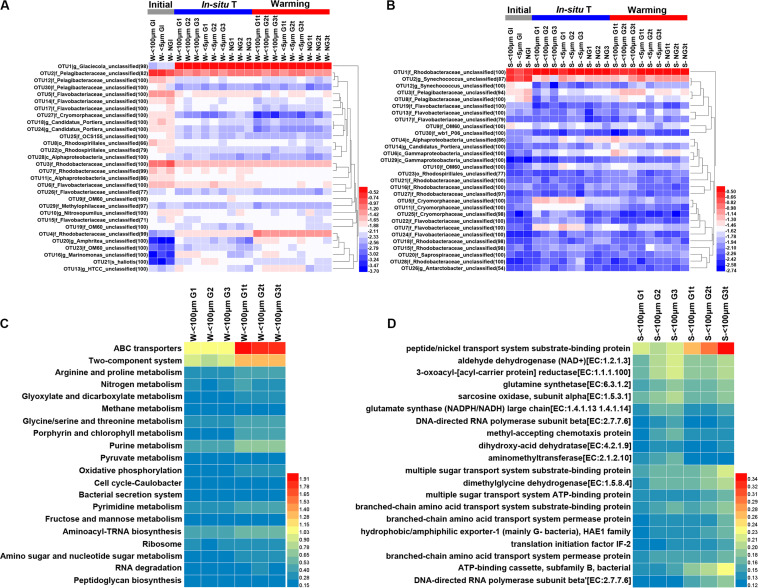
Heatmap exhibiting the relative abundance (transformed by log10) of the top 30 abundant OTUs in each sample during winter **(A)** and summer **(B)** and the percentages (%) of the top 20 KEGG Orthology (KO) categories predicted from 16S rRNA dataset in winter **(C)** and summer **(D)**. Initial, the ambient samples; *In situ* T, incubation in *in situ* temperature; Warming, incubation in the elevated three degrees. <100 μm G, only containing grazers smaller than 100 μm; <5 μm G, only containing grazers smaller than 5 μm; NG, no grazers. W, winter; S, Summer. GI, initial groups; G, groups incubated in *in situ* temperature; Gt, groups incubated in temperature three degrees higher than the *in situ*. Numbers following sample name indicating triplicate samples.

There was a shift from a low relative abundance of OTU4 (affiliated with family Rhodobacteraceae), OTU13 (affiliated with genus *HTCC*) OTU19 (affiliated with family OM60), OTU20 (*Amphrltea sp*.) and OTU23 (affiliated with family OM60) to a high relative abundance in response to the elevated temperature in winter whereas the relative abundance of OTU14 (affiliated with family Flavobacteriaceae), OTU17 (affiliated with family Flavobacteriaceae), OTU7 (affiliated with family Rhodobacteraceae), OTU6 (affiliated with family Flavobacteriaceae), and OTU21 (*hallotis*) decreased (Figure 8A). The presence of grazers resulted in the decline of relative abundance for OTU 12 (affiliated with family Pelagibacteraceae), OTU26 (affiliated with family Flavobacteriaceae), OTU27 (affiliated with family Cryomorphaceae) and OTU29 (affiliated with family Methylophllaceae) but increased that for OTU13 (*HTCC sp*.), OTU16 (*Marinomonas* sp.), OTU18 (*Candidatus Portiera sp*.) and OTU20 (*Amphritea sp*.) in winter (Figure 8A). Interesting, the relative abundance of few OTUs affected by elevated temperature in summer (Figure 8B). Nevertheless, the effect of grazers on the relative abundance of bacteria was still observed (Figure 8B). For example, the relative abundance of OTU3 (affiliated with family Pelagibacteraceae), OTU4 (affiliated with class Alphaproteobacteria), OTU6 (affiliated with class Gammaproteobacteria) were enhanced by the presence of grazers and that of OTU5 (affiliated with family Cryomorphaceae), OTU7 (affiliated with family Flavobacteriaceae), OTU11 (affiliated with family Cryomorphaceae), OTU22 (affiliated with family Flavobacteriaceae), OTU25 (affiliated with family Cryomorphaceae) were weakened (Figure 8B).

Given the different responses of bacterioplankton in family and OTU level to warming, functional profiles were inferred with Tax4Fun to obtain a further understanding of warming effects on bacterioplankton. Obviously, genes involved in ABC transporters and two-component system were more abundant after warming treatment in winter while genes involved in peptide/nickel transport system substrate-binding protein were more abundant after warming treatment in summer (Figures 8C,D).

## Discussion

A predictive understanding of the response of biogeochemical processes to warming in subtropical coastal waters is predicated on knowledge of the critical microbial taxa that dominate those ecosystems. We reported herein the effect of warming in a subtropical zone during summer (September) and winter (December), when *in situ* temperature was 28.5°C and 19.1°C, respectively. Size filtrations were conducted to investigate the influence of grazers of different sizes on the bacterial diversity and community composition. It has been suggested that size filtrations might remove particle associated bacteria which often have higher activity than free-living bacteria (Grossart et al., 2003). Notably, the abundance of free-living bacterioplankton did not change significantly after size filtrations, suggesting the size fractionation method is suitable for evaluating grazing pressure of free-living bacteria in Hong Kong waters.

### Effect of Grazing on Bacterial Abundance and Community Composition

A large number of studies have focused on the factors affecting bacterial abundance and community composition in recent years (Weinbauer et al., 2007). Grazing is a major factor of controlling bacterial standing stock (Sherr and Sherr, 2002; Tsai et al., 2015). The current study found the significant decrease of bacterial abundance as a result of the presence of grazers, especially with grazers less than 5 μm in summer (Supplementary Figure S1 and Table 1). This finding suggests that compared with grazers >5 μm, grazers <5 μm were more efficient bacterivores at this site in summer. It is consistent with many previous studies that smaller grazers are major bacterivores (Unrein et al., 2007; Tsai et al., 2011).

On the other hand, several studies have found that the presence of grazers can affect bacterial community composition in offshore Mediterranean (Bonilla-Findji et al., 2009) and a canyon-shaped reservoir in South Bohemia, Czech Republic (Weinbauer et al., 2007), although using the method of denaturing gradient gel electrophoresis (DGGE). However, in the present study, ANOSIM tests exhibited there was no significant difference of bacterial community composition between individual two groups (Table 2). But we still observed some changes of bacterial relative abundance in response to grazing at family and OTU levels (Figures 5, 8). For example, the presence of >5 μm grazers enhanced the relative abundance of Microbacteruaceae, C111, Marin group II, etc. but weakened that of Kordiimonadaceae and unclassified affiliated with Proteobacteria in winter (Figure 5A). It could be owing to the selective grazing of grazers on bacteria according to their size, surface characteristics and motility (Sanders, 1988; Monger and Landry, 1991; Posch et al., 2001).

### Effect of Warming on Bacterial Abundance, Growth, and Grazing

Rising water temperature caused by water warming is considered to be a powerful driving factor for changing the bacterial abundance (Morán et al., 2015; Arandia-Gorostidi et al., 2017), further affecting oceanic biogeochemical cycles (Sarmiento et al., 2004). Our experimental data showed that warming had significantly positive effect on bacterial abundance in winter (Supplementary Figure S1 and Table 1). Similar result was observed in a similar warming experiment by increasing the temperature up to five degrees compare to the ambient temperature conducted in a fjord in Norway (Lara et al., 2013). However, this phenomenon did not show in the summer which is consistent with another study performed in a subtropical coastal water (Ha Long Bay in Viet Nam) where ambient temperature was around 30°C (Tuyet et al., 2015). In addition to temperature, bacterial dynamics is considered to be affected by resource availability (Murrell, 2003). In the sampling station, the concentration of total organic carbon (TOC) and ammonium were high in December than that in September (Supplementary Figure S3), and the highest total inorganic nitrogen (TIN) and phosphate concentrations occurred in winter while there is a nutrient depletion in summer (Yung et al., 2001; Xia et al., 2015b). This may result in bacterial abundance difference responding to warming between winter and summer. Another possible reason is that the temperature of subtropical waters in summer has reached the optimal temperature (*T*_opt_) of many bacterioplankton taxa where warming would not promote the growth of bacterioplankton any more. It is in an agreement with the study conducted in Hong Kong waters which found a decreased sensitivity of phytoplankton growth rate to temperature in subtropical environments (Liu et al., 2018). In addition, a positive effect of warming on bacterioplankton grazing rates has been observed in several studies focusing on the middle and high latitude waters (Rose et al., 2008; Tsai et al., 2015; Šolić et al., 2017, 2018). In this study, warming only increased the grazing rates of <100 μm grazers in winter and had no effect in summer (Figure 1) which suggested that warming would promote transfer rate of carbon from dissolved organic carbon to bacterioplankton to lager grazers and further affect the biogeochemical cycles in subtropical waters with higher annual average temperature during winter. Together, our results suggested that warming has stronger impacts on growth, abundance and grazing of bacterioplankton in subtropical waters in winter.

### Effect of Warming on Bacterial Community Composition and Functional Profiles

In the current study, ANOSIM tests exhibited no significant difference of bacterial community composition between *in situ* incubation groups and elevated three degrees incubation groups in both seasons (Table 2) and bacterial phylum showed little responses to warming (Figure 3). It is different from the mesocosm studies conducted in Baltic Sea, a temperate water, where found warming may result in important changes of bacterial community composition (Lindh et al., 2013; Bergen et al., 2016). Nevertheless, bacterioplankton had different responses to warming in family and OTU level (Figures 6, 8). For example, the relative abundance of Rhodobacteraceae was significantly enhanced by warming while that of Pelagibacteraceae showed negatively responses to warming without grazers in winter (Figure 6). Rhodobacteraceae, a group associated with sulfur and carbon biogeochemical cycling (Pujalte et al., 2014), showed higher activation energy which suggesting an advantage to outcompete other groups under warming conditions (Arandia-Gorostidi et al., 2017). However, Bergen et al. (2016) found Rhodobacteraceae had higher relative abundance in cold treatment. The contradiction probably results from the high diversity of the Rhodobacteraceae, which includes representatives of the ubiquitous Roseobacter clade as well as species - occupied several ecological niches in various habitats (Buchan et al., 2005; Brinkhoff et al., 2008). Warming significantly increased the relative abundance of Rhodobacteraceae. Rhodobacteraceae can utilize various carbon sources even carbon monoxide (CO) and aromatic compound (Wagber-Döbler and Biebl, 2006) which could not be used by most bacteria, and then warming may change the structure of marine carbon cycle. ABC transporters which can mobilize a variety of substrates across the cell membrane, such as amino acids, nucleotides, metal clusters, lipid molecules, and oligonucleotides play crucial roles in bacterioplankton in oligotrophic environment (Wilkins et al., 2013). Therefore, more carbon in the environment would be transferred to bacterioplankton under warming condition in winter. On the contrary, Pelagibacteraceae showed little responses to warming except the no grazers group in winter probably because they are low temperature-sensitivity populations due to their streamlined genomes (Giovannoni et al., 2005; Grote et al., 2012). A previous warming experiment conducted in Baltic Sea found that two Pelagibacteraceae-related OTUs were temperature sensitive (Lindh et al., 2015). They increased the temperature by 10 degrees in their experiment while we only increased 3 degrees according to the global warming model prediction (Rogelj et al., 2010). In summary, bacteria within the communities had different temperature optima (Lindh et al., 2013). Studying the response of bacteria at family or even OTU level to global warming could achieve more effective information about specific bacterial taxa with different functions and get more accurate prediction on marine biogeochemical cycle in the future.

It should be noted that *Glaciecola sp*. which had an ecological niche during diatom blooms at low temperature (von Scheibner et al., 2017) was the dominant OTU of the experiment groups in winter. Phytoplankton at PM7 was dominated by diatoms except in August (Liu et al., 2010) and *Glaciecola sp.* was a dominant consumer of diatom-derived DOC at low water temperature (von Scheibner et al., 2017). However, *Glaciecola sp.* showed little responses to warming. In addition, the fast growth of opportunistic bacteria (Alteromonadaceae), together with a decrease of the oligotrophic Pelagibacteraceae (Figure 3C) in winter experiment may reflect the bottle effect (Massana and Jürgens, 2003). Whether the changes of the relative abundance of specific bacterial taxa between the initial and after incubations, in both winter and summer (Figures 8A,B), had something to do with the bottle incubation should be noticed.

## Conclusion

This study conducted in the Hong Kong waters could provide insights into the seasonal effect of warming on bacterioplankton in subtropical coastal waters, with higher annual average temperature and distinct seasonal characteristics. In general, our study revealed that there were seasonal differences in the effects of warming on bacterioplankton in subtropical waters. Therefore, when studying the impacts of warming, the waters *in situ* temperature is critical. Lower the waters *in situ* temperature is, stronger the warming effects are. In addition, growth rate of bacteria and grazing strengthened by warming in winter may suggest that warming would promote transfer rate of carbon from dissolved organic carbon to bacterioplankton to lager grazers and further affect the biogeochemical cycles even in subtropical waters. Although bacterial phylum showed little responses to warming in short-term incubations, changes in family and OTU level clearly occurred, with some specific bacterial taxa significantly benefited from or inhibited by increasing water temperature. Hence, long term warming may eventually result in changes of microbial population and function.

## Data Availability Statement

Publicly available datasets were analyzed in this study. This data can be found under accession number: PRJNA596599.

## Author Contributions

XX conceived and designed the study. CL and XX performed the experiments. BG and XX analyzed the data. BG and XX wrote the manuscript. BG, YT, HL, and XX reviewed and edited the manuscript. All authors contributed to the article and approved the submitted version.

## Conflict of Interest

The authors declare that the research was conducted in the absence of any commercial or financial relationships that could be construed as a potential conflict of interest.
