# Supplementary figures and images for: Effect of Warming on Growth, Grazing, and Community Composition of Free-Living Bacterioplankton in Subtropical Coastal Waters During Winter and Summer
Source: Front Microbiol. 2020 Oct 6;11:534404. doi: 10.3389/fmicb.2020.534404 (PMC7573218; doi:10.3389/fmicb.2020.534404)

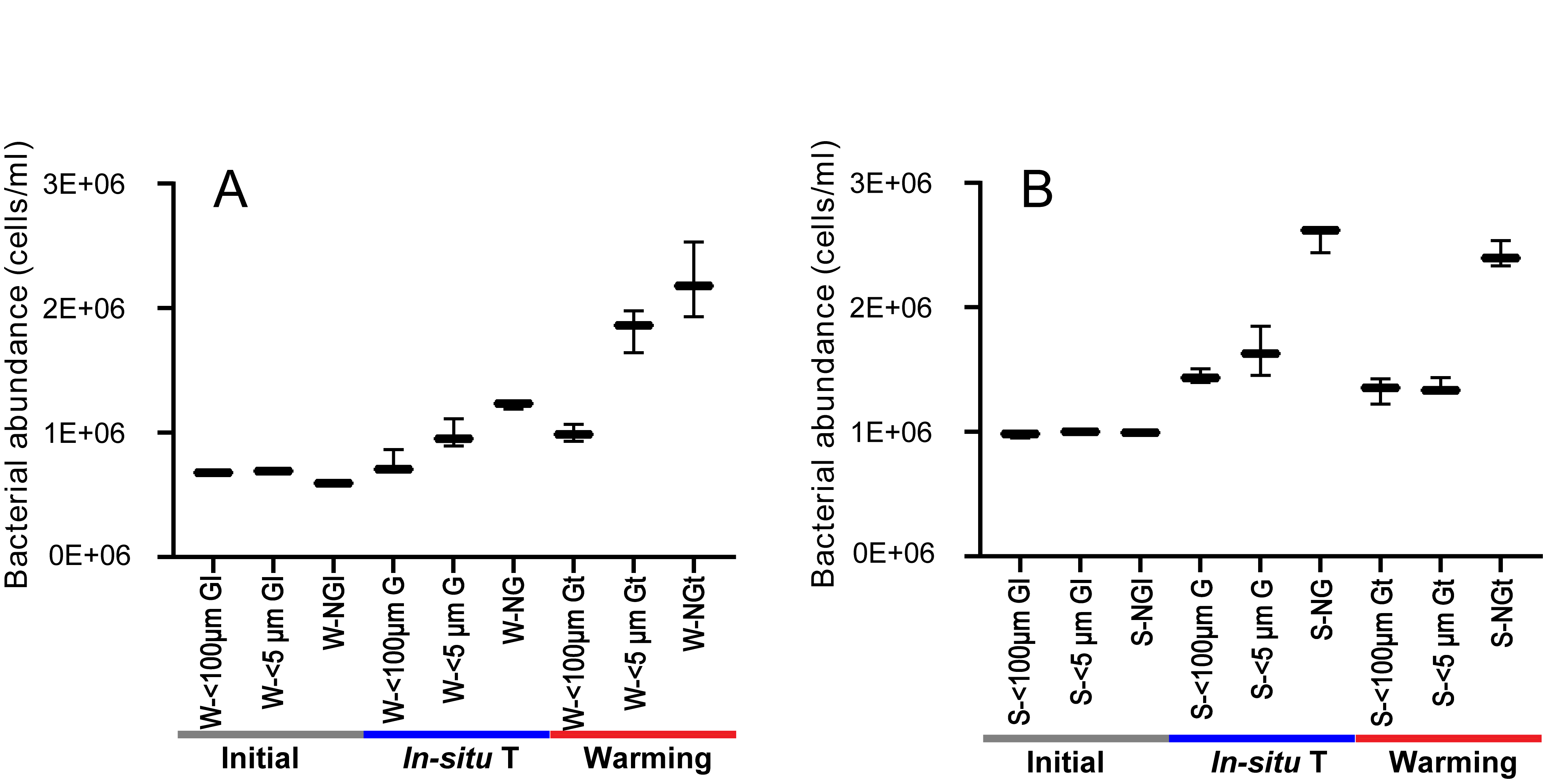

Supplement: Supplementary file 1 [file Image_1.TIF]

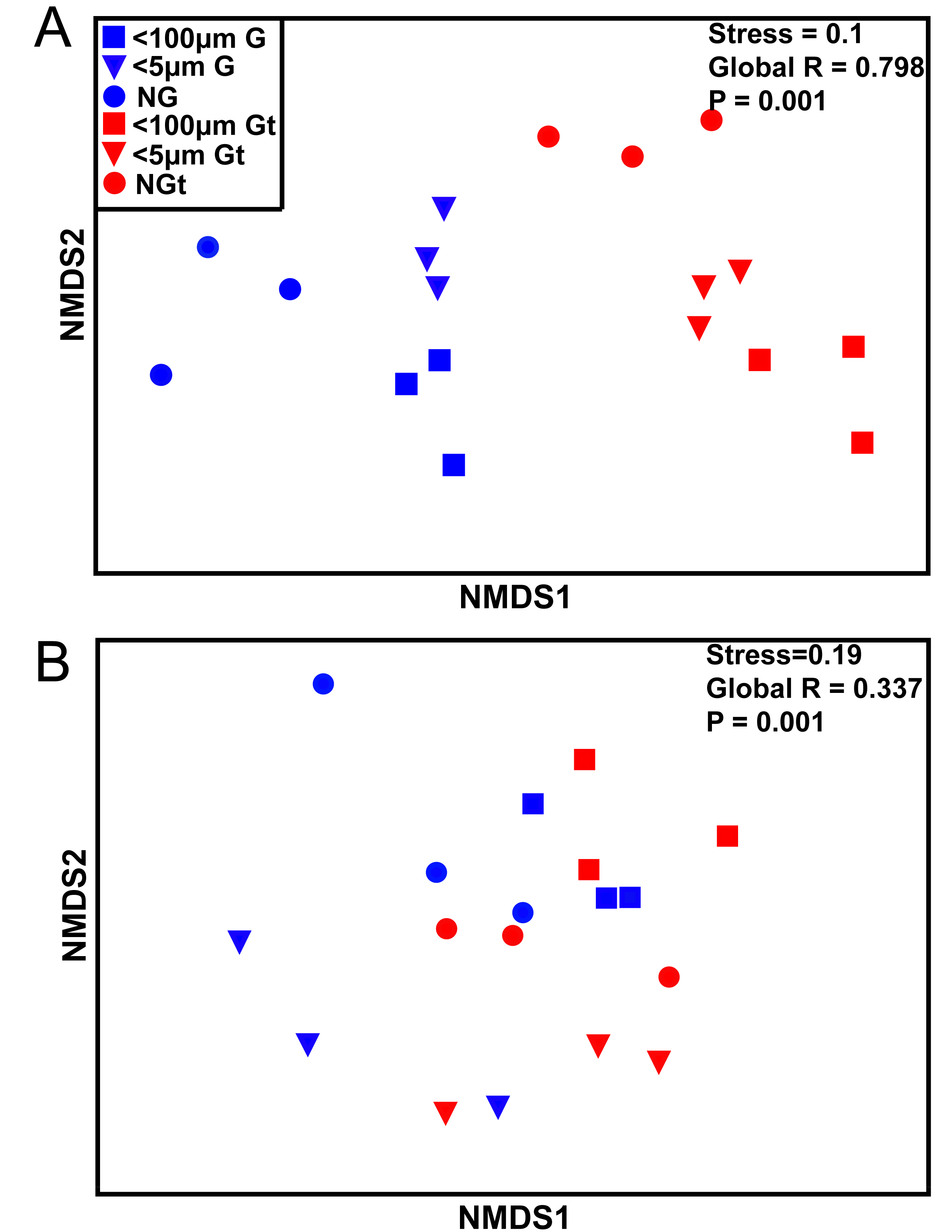

Supplement: Supplementary file 2 [file Image_2.TIF]

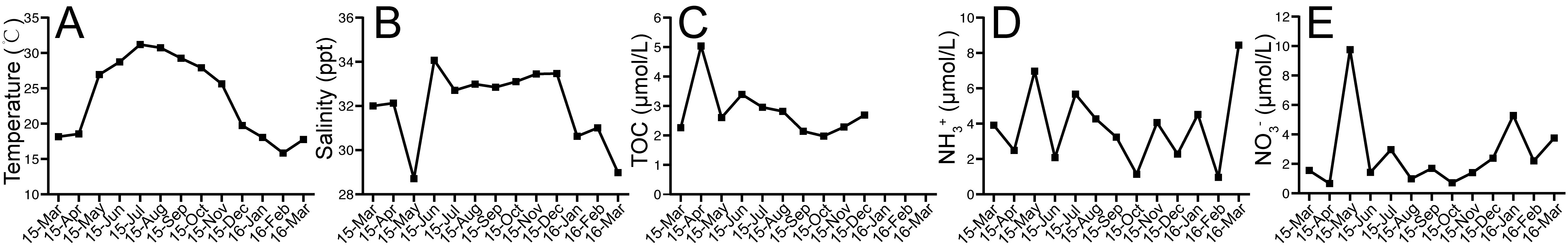

Supplement: Supplementary file 3 [file Image_3.TIF]
